# An Overview of Brain-Derived Neurotrophic Factor and Implications for Excitotoxic Vulnerability in the Hippocampus

**DOI:** 10.1155/2011/654085

**Published:** 2011-09-28

**Authors:** Patrick S. Murray, Philip V. Holmes

**Affiliations:** ^1^Neuroscience Program, Biomedical and Health Sciences Institute, The University of Georgia, Athens, GA 30602, USA; ^2^Behavioral and Brain Sciences Program, Psychology Department, The University of Georgia, Athens, GA 30602, USA

## Abstract

The present paper examines the nature and function of brain-derived neurotrophic factor (BDNF) in the hippocampal formation and the consequences of changes in its expression. The paper focuses on literature describing the role of BDNF in hippocampal development and neuroplasticity. BDNF expression is highly sensitive to developmental and environmental factors, and increased BDNF signaling enhances neurogenesis, neurite sprouting, electrophysiological activity, and other processes reflective of a general enhancement of hippocampal function. Such increases in activity may mediate beneficial effects such as enhanced learning and memory. However, the increased activity also comes at a cost: BDNF plasticity renders the hippocampus more vulnerable to hyperexcitability and/or excitotoxic damage. Exercise dramatically increases hippocampal BDNF levels and produces behavioral effects consistent with this phenomenon. In analyzing the literature regarding exercise-induced regulation of BDNF, this paper provides a theoretical model for how the potentially deleterious consequences of BDNF plasticity may be modulated by other endogenous factors. The peptide galanin may play such a role by regulating hippocampal excitability.

## 1. Brain-Derived Neurotrophic Factor Overview

Brain-derived neurotrophic factor (BDNF) is a member of the neurotrophin family, a group of structurally related polypeptide growth factors. The family also includes nerve growth factor (NGF), neurotrophin-3 (NT-3), and neurotrophin-4 (NT-4/5) [1]. Other neurotrophins have also been identified, NT-6 and NT-7; however, these likely do not exist in mammals [2–4]. Neurotrophins activate one or more of the high-affinity tropomyosin-receptor kinase (Trk) receptor family [1, 5, 6], as well as the low-affinity p75 neurotrophin receptor (p75NTR) [7]. 

Neurotrophins direct growth and differentiation in the developing nervous system [1, 3, 6, 8]. Levels of the different neurotrophins relate in a predictable pattern to stages of embryonic development. Infusion of BDNF into the adult brain promotes neurogenesis [9, 10], and dendritic spine reorganization in the rat hippocampal formation [11]. BDNF gene transfection triggers dendritic and axonal branching in dentate gyrus (DG) granule cell cultures [12] while mice with a targeted gene deletion show substantial impairment in normal cerebellar development, among other developmental and behavioral deficits [13].

Trk receptors precipitate the activation of many signaling cascades, including phospholipase C (PLC), phosphoinositide 3-kinase (PI3K), and Ras [14, 15], which are involved in different aspects of neuronal survival [16] and neurite outgrowth [17–19]. The p75NTR is implicated in both pro- and antitrophic processes, such as neurite outgrowth [20] and apoptosis [14, 21, 22].

BDNF regulates synaptic transmission and activity-dependent plasticity [23, 24] and promotes long-term potentiation (LTP) [25]. Its effects on LTP occur through both pre- and postsynaptic mechanisms [26]. BDNF exerts presynaptic effects in a retrograde or paracrine fashion [27, 28] and postsynaptic effects by modulating NMDA receptor subunit expression [29]. Experimental reductions in BDNR or TrkB activity significantly impair LTP [25, 30, 31].

Previous research has demonstrated that exercise increases hippocampal BDNF mRNA [32, 33] and protein [34, 35]. The effect of exercise to augment BDNF activity depends on cAMP-response-element binding protein (CREB) activation, as CREB suppression mitigates this effect [36]. Increases in hippocampal BDNF mRNA have been detected after a period of running as short as 6 hours [37], to as long as two months [38], signifying a sensitive yet sustainable effect. Physical activity leads to greater activation of cellular signaling pathways associated with survival [39] and neurogenesis [40], and can enhance hippocampal LTP induction [32], all of which strongly implicate BDNF in their effects.

This review will describe how BDNF and neurotrophins are involved in nervous system development, the signal transduction mechanisms associated with neurotrophin activity, and the modulating role BDNF plays in synaptic plasticity and LTP. The influence of exercise on BDNF activity, especially in the hippocampus, and the relationship between BDNF and excitotoxicity will also be examined.

## 2. Signal Transduction

**Figure 1:**
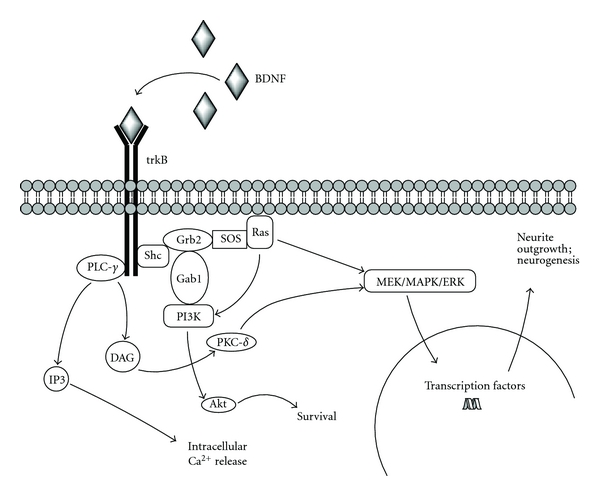
TrkB transduction mechanisms. Aspects adapted from [1, 15].

BDNF and NT-4/5 bind to TrkB receptors [41, 42], as does NT-3 to a lesser extent. NGF binds to TrkA receptors, and NT-3 binds to TrkC receptors [5]. When a ligand binds to a Trk receptor (see Figure 1), the receptor dimerizes and autophosphorylates tyrosine residues to yield docking sites for the src homology 2 domain-containing adapter protein (Shc) [43] and phospholipase C-*γ* (PLC-*γ*), which are coupled to intracellular signaling cascades such as Ras [44], PI3K [45, 46], and, as mentioned, PLC-*γ* [14, 15, 47, 48]. 

Shc regulates protein interactions by binding, with a high-affinity, tyrosine-phosphorylated sites, and in this way regulates intracellular signaling such as Ras [43]. Once Shc is docked with the receptor and bound to the adapter protein Grb2, Ras promotes activation of the mitogen-activated protein kinase (MAPK)/extracellular signal regulated kinase (ERK) cascade, as well as the PI3K cascade [3, 15]. Ras is linked to Grb2 by the guanine nucleotide releasing factor SOS [49]. MAPK/ERK is essential for neurogenesis [50] and promotes survival by two ways, by induction of prosurvival genes and inhibition of proapoptotic proteins [51]. Ras also suppresses apoptosis via PI3K [16]; PI3K activates Akt, which sequesters pro-apoptotic proteins in the cytoplasm away from their transcriptional targets [52].

The PI3K cascade can be activated two ways by Trk receptors, through Ras as well as through adapter proteins Shc, Grb2, and Grb2-associated binder-1 (Gab1) [15, 45]. In some neurons, BDNF phosphorylates the insulin receptor substrate, which can activate PI3K as well [46]. Vaillant and colleagues [16] demonstrated that inhibition of any portion of the Ras-PI3K-Akt pathway significantly reduced survival of sympathetic neurons in culture in the presence of NGF.

The adapter proteins Shc and PLC-*γ* are phosphorylated once they dock with the Trk receptor [3, 14, 47]. Once activated, PLC-*γ* then catalyzes the breakdown of lipids to inositol 1,4,5 triphosphate (IP3), which promotes the release of calcium from intracellular stores, and a subsequent increase in intracellular calcium concentration and diacylglycerol (DAG) occurs [48]. DAG regulates protein kinase C (PKC-*δ*), which may be required for the MAPK/ERK signal [17], and both DAG and MAPK/ERK have been implicated in neurite outgrowth [18, 19]. Interestingly, LTP is inhibited when PLC-*γ* signaling is blocked [26], which may be related to reduced IP3 formation and subsequent reduction in intracellular calcium release.

The other neurotrophin-responsive receptor, the p75NTR, can bind to each factor and regulate the Trk receptor affinity for its ligand [53]. The Trk receptors and the p75NTR are often present in close proximity on the cellular membrane [54]. While TrkB responds to NT-3, NT-4/5, and BDNF [5], the receptor selectively binds only BDNF when colocalized with p75NTR [54]. The largely presynaptic p75NTR [55] serves dual roles in that it modulates Trk receptor binding and is involved in prolonged Ras-mediated activation of ERK and neurite outgrowth [20], though p75NTR-induced neurite outgrowth ceases after prolonged p75NTR activation [20]. The p75NTR also activates c-*jun* N-terminal kinase (JNK) [56] and causes apoptosis in a variety of neurons [14, 21, 22]. 

Although all Trk receptors are assumed to behave in much the same fashion, differences do exist [53]. Kaplan and Miller [14] demonstrated, using TrkB receptors with mutations at different binding sites, that TrkB receptors use both PI3K and MAPK cascades for cell survival while TrkA receptors depend mainly on PI3K. This demonstrates some comparative flexibility for TrkB receptors with regard to pro-survival function. Importantly, two truncated forms of TrkB receptors exist that lack tyrosine kinase function [57] and bind BDNF to negatively modulate TrkB activity [58].

## 3. Development

Given that BDNF-associated signaling modulates neuron survival and apoptosis, much attention has been focused on the role of BDNF (and other neurotrophins) in development. Indeed, the critical involvement of Trk receptors in neuronal survival [16, 50–52], neurogenesis [10], and neurite outgrowth [12, 18, 19] implicates BDNF in developmental processes. Throughout development, neurotrophins influence and guide neuronal differentiation in the central nervous system (CNS) [6, 8] and peripheral nervous system (PNS) [1, 3]. Barnabé-Heider and Miller [50] demonstrated that cortical progenitor cells express BDNF and NT-3 and their associated TrkB and TrkC receptors, and that these neurotrophins were essential to the survival of the progenitor cells. 

BDNF mRNA expression is lowest in the brains of early rat embryos and increases into adulthood [59]. It demonstrates a reciprocal relationship with NT-3 mRNA, which is at the highest levels in early embryos. Maisonpierre and colleagues [59] characterized distribution of BDNF, NT-3, and NGF mRNA during rat development and found that the amount of all three dramatically increased between embryonic days 11 and 12, with transcripts widely distributed by embryonic day 13. This timing corresponds directly to the onset of robust neurogenesis in the peripheral and central nervous systems [60, 61]. BDNF transcript levels continued to increase into adulthood, especially in the hippocampus [59].

Katoh-Semba et al. [62] examined BDNF protein concentration during postnatal development in rats and found that levels in the septum, cerebellum, and hippocampus increased over time and then stabilized (although hippocampal protein levels took much longer to plateau) while hypothalamic BDNF protein levels increased for a period following birth and then decreased. Kim et al. [63] further investigated BDNF-immunoreactivity (IR) in the rat forebrain and upper brainstem during postnatal development. The investigators defined three groups of BDNF cells: those that are detected early, increase in number, and remain stable during adulthood (e.g., piriform cortex, cingulate gyrus), those that increase with age then decrease in adulthood (e.g., basolateral nucleus of the amygdala), and those that appear briefly before dramatic reductions in adulthood (e.g., substantia nigra, various hypothalamic nuclei). 

In the hippocampus, BDNF-IR cells appeared in the pyramidal layer of CA2 and CA3 early on (postnatal day 7) and staining increased through 28 days following parturition but then decreased, with comparatively reduced cell staining detected in 56-day-old adults [63]. The BDNF-IR cell decrease detected in CA2 and CA3 in adulthood was completely absent in colchicine-treated adults, which instead showed an increase in BDNF-IR cells in these areas, possibly reflecting increased accumulation of BDNF in the cell body associated with inhibited axonal transport. BDNF-IR fibers were detected in the stratum lucidum layer of CA2 and CA3 and the polymorph layer of the DG also early on during the intial 28-day postnatal period, and these BDNF-IR fibers actually continued to increase into adulthood [63]. 

BDNF serves a unique role among neurotrophins in development, especially with regard to the cerebellum. An examination of the cerebella of mice with a targeted BDNF gene deletion (BNDF^−/−^ mice) revealed that BDNF is essential for normal development and function of the cerebellar cortex [13]. The study showed reduced Trk activation in Purkinje cell bodies and dendrites in the mutant mice, indicating a lack of redundancy among neurotrophins with regard to BDNF in these cells. In BNDF^−/−^ mice, there was a dramatic increase in cell death among developing granule cells, impaired development of the layers of the cerebellar cortex, and irregular foliation in the middle and posterior cerebellum; defects were apparent in the declival sulcus (absent), the intercrural fissure (absent), the prepyramidal fissure (nearly absent), and uvular sulcus (nearly absent) [13].

BNDF is crucial to postnatal survival, as most BNDF^−/−^ mice die shortly after birth, but some do survive for a month or more [64, 65]. Mice that lack BDNF demonstrate severe deficiencies in PNS development, especially with regard to afferent neurons, but exhibit comparatively mild deficiencies in CNS development [64, 66]. BNDF^−/−^ mice show an abnormal gait with uncoordinated movements, and their stance is substantially wider (distance between left and right paws), despite their smaller size compared to wildtype mice [13]. This evidence further implicates the importance of BDNF not just to neuron development in the PNS but also to cerebellar development and function.

Intraventricular BDNF application encourages neurogenesis in several parts of the rat brain, such as striatum, septum, thalamus, and hypothalamus [9], and infusion directly into the hippocampus increases the number of granule cells in the DG [10]. Danzer and colleagues [12] transfected hippocampal cells in culture with either BDNF or NGF; DG granule cells exhibited considerable axonal and dendritic branching following BDNF, but not NGF transfection. This effect was abolished with the application of a Trk receptor tyrosine kinase inhibitor, demonstrating that BDNF and Trk signaling promote neurogenesis both within and outside of the context of development.

**Figure 2:**
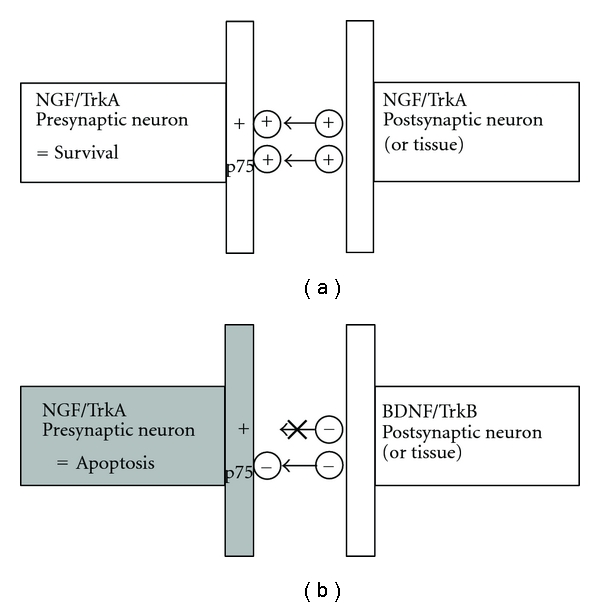
Neurotrophin signal involvement in targeting of growing neurons. (a) Both TrkA and p75NTR are activated on the growing neuron, survival and growth continues; (b) on the growing neuron, p75NTR is activated by BDNF, but no TrkA activation takes place, so apoptosis occurs. Adapted from [67].

The involvement of p75NTR in refining Trk receptor selectivity may serve to modulate targeting of growing neurons (see Figure 2), with neurotrophic factors acting as markers [67]. Target tissues for growing PNS neurons (e.g., skeletal muscle) express neurotrophins [68], and BDNF is released postsynaptically to influence CNS neurons in a retrograde fashion [27, 69]. In a study of rat sympathetic neurons that largely express NGF and TrkA receptors, BDNF activated p75NTR-mediated apoptosis in conditions with low available NGF [70]. For example, if a predominantly NGF/TrkA-associated neuron were to grow to network with a predominantly BDNF/TrkB-associated neuron, the growing neuron would quickly become apoptotic. Postsynaptic BDNF release and activation of the presynaptic p75NTR [55], coupled with the lack of NGF/TrkA signaling, would reduce the survivability of the growing neuron. Thus p75NTR serves a regulatory role with regard to Trk receptor behavior and neuronal proliferation.

In summary, neurotrophins influence neuronal differentiation [10] and are important to neuronal survival [16, 50–52]. BDNF mRNA is present in relatively low levels early in life and generally increases through to adulthood [59, 62], especially in certain hippocampal neurons [63]. Studies of mice lacking BDNF demonstrate how it is essential for proper PNS [64, 66] and cerebellar development [13].

## 4. Synaptic Plasticity

**Figure 3:**
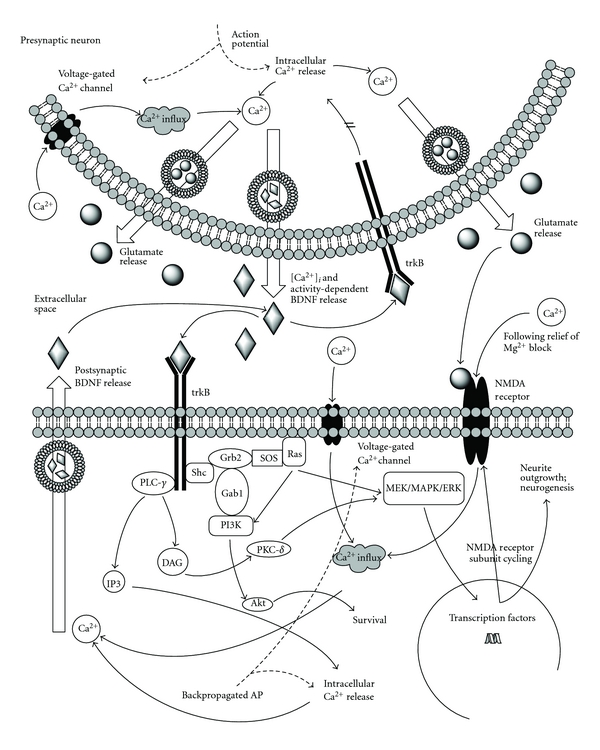
BDNF involvement in the induction of LTP and increased neuronal excitability. Aspects adapted from [1, 15].

BDNF is directly involved in the regulation of synaptic transmission and activity-dependent synaptic plasticity by both pre- and postsynaptic mechanisms [1, 23] (see Figure 3). These mechanisms underlie the critical role of BDNF in LTP [24–26, 29, 65]. In a study with cultured hippocampal and cerebellar granule cells, Caldeira and colleagues [29] demonstrated that BDNF increased the levels of NMDA receptor subunits in the plasma membrane of hippocampal cells. They recorded a correlated increase in intracellular calcium concentration and described an increased calcium entry through the additional NMDA receptors. Interestingly, Hartmann and colleagues [69] stimulated cultured hippocampal neurons presynaptically and detected a postsynaptic BDNF release, dependent upon calcium influx, thereby suggesting a BDNF-dependent positive feedback loop between presynaptic and postsynaptic signaling in the generation of LTP.

Walz et al. [28] found that BDNF is essential for activity-induced potentiation of presynaptic vesicle cycling in cultured embryonic neocortical neurons of BDNF-knockout mice. This phenomenon was dependent upon NMDA receptor activation, and was significantly reduced in BDNF-deficient neurons, indicating a possible retrograde messenger or paracrine role for BDNF. In support of these findings, previous work in BDNF knockout mice [65] showed a pronounced impairment of vesicle docking in hippocampal synapses while direct application of BDNF reversed the deficits. Estrogen promotes hippocampal plasticity, and in ovariectomized rats, the loss of estrogen reduces cytoskeletal reorganization in hippocampal dendritic spines and produces deficits in LTP [11]. Kramár and colleagues recently demonstrated that application of BDNF to hippocampal sections restored spine actin polymerization and LTP stability in these rats [11].

Backpropagating (retrograde) action potentials modulate short- and long-term changes to synaptic activity [71] and also serve to relieve the Mg^2+^ block on NMDA receptors [72]. They are also sufficient to trigger a dendritic release of BDNF [27]. The extent of backpropagation in a neuron is dependent upon dendritic morphology, ion channel distribution, and synaptic input [71]. Importantly, application of an endoplasmic reticulum Ca^2+^-ATPase inhibitor stops this dendritic BDNF release [27], suggesting a calcium-dependent mechanism for retrograde release. These findings thus reveal several mechanisms for BDNF-mediated promotion of LTP in the hippocampus [1].

BDNF is released in an activity-dependent manner, as demonstrated in both hippocampal neurons [73] and peripheral neurons [74]. While it may not be the only mediator of LTP, mice lacking BDNF demonstrated reduced tetanus-induced LTP [30], a deficit that disappeared with the return of BDNF function [31]. Similarly, rats with a reduction in TrkB receptors showed reduced LTP induction [25]. As described above, BDNF works through both pre- and postsynaptic mechanisms to support LTP, and a simultaneous block of pre- and postsynaptic PLC-*γ* activity, but not either by itself, is required to reduce LTP in mouse hippocampal cultures [26]. Interestingly, the involvement of TrkB in LTP is not completely dependent on BDNF signaling; NMDA receptors can activate TrkB by way of zinc influx, and adenosine 2A receptors can also influence TrkB [24].

In brief, BDNF is involved in trafficking NMDA receptor subunits to the membrane, thereby increasing the potential for calcium influx [29]. Backpropagating action potentials also influence calcium influx by opening voltage-dependent calcium channels [27]. A sufficient local buildup of calcium causes postsynaptic BDNF release [69], which then leads to an increase in presynaptic vesicle cycling [28]. These mechanisms all serve to support LTP and synaptic plasticity.

## 5. Exercise

Exercise leads to substantial changes in BDNF and NMDA receptor activity in the hippocampus, and these changes in large part underlie the effects of exercise on learning and synaptic plasticity. In rats with free access to activity wheels for one to six weeks, increases in hippocampal BDNF mRNA [32, 33] and BDNF protein [34, 35] have been detected. These increases in BDNF remain for some time after cessation of exercise as well. Berchtold, Castello, and Cotman demonstrated that hippocampal BDNF protein remained elevated for two weeks following three weeks of access to cage wheels (protein levels were 186% of controls at the end of exercise, 137% one week followup, and 133% at two weeks) [75]. 

Physical activity increases hippocampal BDNF expression after a relatively short amount of time, as voluntary wheel running induced BDNF mRNA after only 6 hours of running [37]. A substantial increase in BDNF mRNA in the DG has also been described after 2 weeks [39], 3 weeks [33], 4 weeks [32], and 2 months of voluntary running [38]. BDNF mRNA and protein induction due to exercise varies over the life-span, however. Younger mice (2 months) exhibit a larger increase compared to older mice (15 and 24 months) over four weeks of running [34]. Forced treadmill running for 30 minutes per day for six weeks increased hippocampal BDNF protein and BrdU positive proliferating cells in younger rats (5 months), an effect that was less robust but still significant in older rats (24 months) [76].

Exercise influences BDNF even under conditions of homeostatic perturbations such as stress or dysfunction in energy metabolism. In chronically stressed Sprague-Dawley rats, access to cage wheels for six weeks only during dark cycle (active period) yielded increased levels of BDNF protein in the striatum compared to sedentary rats [77]. The characteristic lower levels of hippocampal BDNF protein and dendritic spine density in type II diabetic mice (*db/db*) were significantly enhanced with free access to cage wheels [78]. Under a condition of food deprivation every other day, six weeks of wheel running still yielded significantly increased hippocampal BDNF protein compared to normally fed and food-deprived sedentary Wistar rats, similar in magnitude to the levels observed in normally fed exercising rats [79].

Running is associated with a robust activation of survival pathways and vesicular proteins, effects that are linked to BDNF. Shen and colleagues [40] showed prolonged hippocampal MAPK signaling following only a week of voluntary running. Chen and Russo-Neustadt [39] demonstrated a significant increase in hippocampal PI3K protein in rats after two weeks of wheel running. In another study, Chen, and Russo-Neustadt found a similar consequence of running on Akt protein, an effect dependent upon CREB signaling [36]; in fact, the researchers showed that exercise-induced increases in hippocampal BDNF protein and mRNA are also CREB activation dependent, indicating the essential nature of CREB signaling to exercise and BDNF-dependent cellular survival effects. These pathways are likely directly associated with the neurogenesis detected in the DG of exercising rats [80, 81]. 

Voluntary exercise selectively increases hippocampal synapsin I and synaptophysin mRNA [35, 82], a phenomenon blocked by a recombinant human TrkB-IgG chimera, a highly specific antagonist of BDNF [83]. Synapsin I tethers vesicles to the cytoskeleton [84] while synaptophysin may modulate vesicle recycling during high-intensity activity [85]. Indeed, this BDNF-mediated increase in synapsin following exercise may in part explain how exercise enhances LTP, along with the other mechanisms for BDNF-facilitated LTP described above. Running rats show a lower threshold for LTP induction in the DG [32]. Voluntary running also yielded better performance on the Morris water maze test [81], an LTP-associated effect abolished with the selective blocking of BDNF [86]. Importantly, Farmer and colleagues [32] also identified an increase in NMDA receptor subunit NR2B mRNA in the DG following activity wheel running, which may represent yet another mechanism explaining how exercise promotes LTP and learning and memory.

## 6. Excitotoxicity

A complicated relationship exists between BDNF and epileptogenic hyperexcitability, but this relationship may be fundamentally characterized as a positive feedback loop. In general, enhanced BDNF signaling is associated with increased excitability, presumably through increased synaptic plasticity. As described above, increased neural activity tends to promote BDNF functions. For example, mRNA and protein levels for BDNF are increased in the hippocampus after seizure [66, 87], and BDNF knockout mice demonstrate reduced seizure behavior in response to kindling [88]. In transgenic mice with an overexpression of the truncated TrkB receptor, the reduced BDNF signaling led to reduced seizure activity after systemic kainic acid administration [58]. Koyama et al. [89] demonstrated development of hyperexcitable circuits due to abnormal mossy fiber sprouting, in part caused by activity-induced BDNF release and Trk receptor activity. This sprouting did not occur in the presence of a Trk receptor tyrosine kinase inhibitor or anti-BDNF antibody. Such abnormal remodeling is, in part, responsible for dentate hyperexcitability [89, 90].

Glutamatergic excitotoxicity, thought to result from excessive calcium influx via NMDA receptors [91], is a major contributor to neuronal damage associated with anoxia [92], ischemia [93], and seizure [94]. In cultured cerebellar granule cells, high glutamate concentrations cause immediate necrosis [95]. Following withdrawal to normal medium, surviving cells undergo apoptosis over time. In rat hippocampal cell cultures, a high concentration of glutamate caused mainly necrosis while a lower concentration of glutamate led almost exclusively to delayed apoptosis [95].

Glazner and Mattzon [96] evaluated the effect of BDNF application on NMDA receptor subunits, and found that NR1 and NR2A protein levels are increased whereas NR2B levels are decreased; associated effects included increased calcium response to NMDA activation, increased necrosis (cell swelling), and decreased apoptosis (cell shrinkage and chromatin fragmentation). BDNF-mediated enhancement of NMDA receptor functions are consistent with the effects of exercise on increasing NMDA receptor expression described above [32], further supporting the role of BDNF in exercise-enhanced synaptic plasticity. These results further illustrate how BDNF overexpression may lead to hyperexcitable circuitry through multiple mechanisms.

BDNF thus encourages neurogenesis [9, 10], axonal, and dendritic sprouting [89], and NMDA receptor-mediated transmission [96] in a Trk-B-dependent manner [12], which are generally considered to be beneficial effects on neuronal function. However, these same mechanisms may lead to hyperexcitable circuits in the DG [89, 90, 97], which may eventually lead to spreading excitotoxicity. This condition can therefore become a self-sustaining cycle of sprouting and hyperexcitability, fueled in part by activity-dependent BDNF release [69, 73, 74]. In a kainic acid model of temporal lobe epilepsy utilizing transgenic mice, increased TrkB signaling facilitated significantly shorter time to epileptogenesis and more severe epileptiform electroencephalogram activity compared to wild-type mice, and epileptogenesis was delayed in reduced TrkB (truncated form) mice [98].

Based on this evidence for a prolonged state of hyperexcitability coupled with a shorter facilitation resulting from enhanced expression of BDNF or TrkB signaling in the hippocampus, it follows that exercise-induced plasticity could leave the brain more vulnerable to epileptic seizures and excitotoxicity. Some experimental evidence supports this hypothesis. Microinjections of kainic acid into the hippocampi of anaesthetized rats produce more damage in subjects that were provided with running wheels for several weeks compared to sedentary controls [99]. This enhanced excitotoxicity in exercising rats was interpreted by the investigators as a consequence of exercise-induced upregulation of hippocampal BDNF expression. 

On the other hand, a large body of evidence from both clinical studies in humans and basic research in animal models reveals that physical exercise produces a wide range of neuroprotective effects [100–103]. For example, exercise in humans decreases risk of neurological disease and stroke, and it may improve recovery after brain trauma [101]. The brain damage caused by experimental infarct is diminished in exercising rats compared to sedentary controls [103]. Exercise also protects against oxidative stress [101], and the neurotoxic effects of 6-OHDA [104, 105] and MPTP [106]. Since neural hyperexcitability is a mechanism for the injury and death that occurs in all of these neurological insults and/or disorders [107, 108], determining how exercise protects against excitotoxicity should have broad implications for understanding its beneficial effects on the brain. Nonetheless, these conditions are only indirectly linked to excitotoxicity, and a more direct examination of the influence of exercise on hyperexcitability is required to understand the mechanisms for its neuroprotective effects.

In contrast to the findings of Ramsden and colleagues [99] described above, in which exercise led to increased excitotoxicty when kainic acid was injected into the hippocampus of anaesthetized rats, some evidence suggests that exercise may protect against hyperexcitability. Experiments specifically designed to this hypothesis have revealed that exercise protects against kainic acid-induced seizures when studied in awake, freely behaving rats. Reiss et al. [109] reported that three weeks of voluntary exercise decreases the effects of kainic acid on seizure behaviors and hippocampal c-fos expression. Further supporting the hypothesis that exercise diminishes hyperexcitability are similar reports of exercise protecting against experimentally induced seizures using other convulsant manipulations [110, 111]. Exercise also diminishes the excitotoxic effects of systemic domoic acid in mice [112] and reduces the learning deficits caused by systemic kainic acid administration in rats [100].

Taken together, the conflicting findings of exercise effects on hyperexcitability and excitotoxicity suggest that an exercise-induced compensatory mechanism may counter the hyperexcitable state associated with upregulation of BDNF. Importantly, this compensatory mechanism may only function in awake, freely behaving subjects. In the report by Ramsden and colleagues, the exercise-associated increase in neurotoxicity caused by kainic acid injected directly into the hippocampi was observed in anesthetized rats [99]. A complex, polysynaptic protective mechanism may have been eliminated by the anaesthesia. If such a mechanism depended on extrahippocampal circuitry, then it is reasonable to assume that it may not engage in an anaesthetized state.

Though it is likely that a variety of mechanisms exist to counter the hyperexcitable state in the hippocampus caused by BDNF, elucidation of such a mechanism related to exercise should be based on specific criteria. This mechanism should involve some neural factor that (1) is upregulated by exercise, (2) diminishes neural hyperexcitability acutely, and (3) exerts latent neuroprotective effects. Research from this laboratory suggests that hippocampal afferents originating from the noradrenergic locus coeruleus may be a critical component of this mechanism. More specifically, experimental evidence points to the neuropeptide galanin as a critical factor in dampening hippocampal excitability.

Galanin is a 29-30 amino acid neurotransmitter and putative trophic factor that regulates neural activity in several brain structures, most notably the hypothalamus and hippocampus. Galanin coexists extensively with norepinephrine in locus coeruleus neurons [113]. Retrograde tracing/double labeling experiments reveal that the hippocampus receives galaninergic innervation via projections from the locus coeruleus [114]. At least three G-protein coupled galanin receptor subtypes have been confirmed, designated GAL R1-3. Galaninergic transmission is predominantly inhibitory, producing hyperpolarization through increased K^+^ or decreased Ca^2+^ conductance [115].

Several lines of evidence reveal that galanin functions as an endogenous neuroprotective factor for hippocampal neurons [116–118]. This neuroprotection appears to be mediated primarily through the regulation of hippocampal excitability. Infusion of galanin into the hippocampus inhibits seizures provoked by a variety of methods, and the galanin receptor antagonists M-35- and M-40 block the antiseizure effects of galanin [117]. Transgenic galanin knock-out mice show increased susceptibility to kainic acid-induced seizures whereas transgenic mice overexpressing galanin display a resistance to these seizures [119]. Hippocampal electrophysiological activity is similarly exaggerated in the galanin knockouts and decreased in galanin overexpressers [119]. In vitro studies in hippocampal cultures from transgenic mice have confirmed that endogenous galanin diminishes excitotoxicity and apoptosis [116]. 

Previous work from this laboratory has demonstrated that either three weeks of voluntary wheel running or treadmill training increases galanin gene expression in locus coeruleus neurons in rats [120–123]. No exercise-induced changes in neuropeptide-Y or tyrosine hydroxylase were observed in these studies, suggesting that locus coeruleus neurons respond to exercise by specifically upregulating galanin. The critical role of galanin in mediating the protective effects of exercise against convulsant-induced seizures was demonstrated by the administration of the galanin receptor antagonist M-40, which largely blocked the neuroprotection as measured by behavioral seizure severity [109]. Galanin receptor blockade thus decreased the anticonvulsant effects of exercise. These results suggest that exercise-induced upregulation of galanin mediates the enhanced inhibitory tone that may counteract BNDF-mediated hyperexcitability.

## 7. Summary and Conclusion

To conclude, BDNF receptor activity encourages neurogenesis [9, 10], suppresses apoptosis [16], and modulates synaptic activity via a variety of signaling cascades [45, 48, 50]. These effects are beneficial with regard to development [1, 8] and synaptic plasticity [25, 26, 29, 65], but can be harmful in conditions sensitive to hyperexcitability [58].

**Figure 4:**
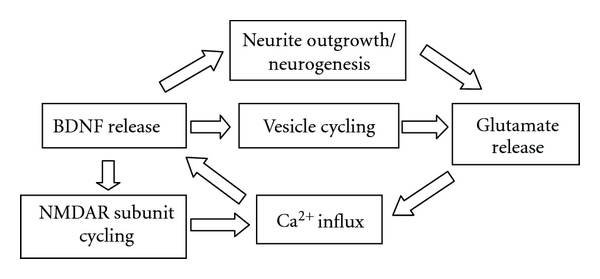
BDNF involvement in the development of hyperexcitable circuits in the hippocampus; although this cycle serves to support LTP and plasticity, it can become overwhelmed under certain excitotoxic conditions and serve to further promote those conditions.

Dendritic BDNF release is activity-dependent, based on calcium influx [27, 69]. This release causes an increase in presynaptic vesicle cycling [28], which further stimulates synaptic activity. The BDNF-induced increase in NMDA receptor subunits [29] creates more opportunity for calcium influx, supporting additional BDNF release. The increased TrkB signaling boosts IP3 activity and intracellular calcium release, contributing to the enhancement of the cycle even further. These processes feed off of one another to overwhelm any modulatory effects of truncated TrkB receptors (which bind BDNF but lack tyrosine kinase function [57]) or p75NTR, the latter of which will act more to refine the actions of the increased available BDNF to yet further potentiate the cycle. Since BDNF encourages neurogenesis [9, 10] and neurite outgrowth [12], the effects of these processes are further compounded with the establishment of additional excitatory synapses (see Figure 4).

Thus hyperexcitable circuits in the DG can develop due to excessive BDNF and NMDA receptor activity [89, 90], activity that maintains a self-supporting cycle of neurotransmitter release in the hippocampus that can be overwhelmed to promote excitotoxic vulnerability. Considering the maladaptive nature of this condition, selection for hippocampal circuitry that regulates this hyperexcitability would provide clear evolutionary benefits. Furthermore, environmental stimuli that promote hippocampal BDNF expression, such as exercise, would be expected to engage this compensatory mechanism. Evidence suggests that a circuit including galaninergic projections from the LC to the hippocampus provides this function [116, 118, 120, 121, 123].
